# Aortitis Increases the Risk of Surgical Complications and Re-Operations After Major Aortic Surgery

**DOI:** 10.3390/jcdd11120405

**Published:** 2024-12-17

**Authors:** Edward Staniforth, Shirish Dubey, Iakovos Ttofi, Vanitha Perinparajah, Jasmina Ttofi, Rohit Vijjhalwar, Raman Uberoi, Ediri Sideso, George Krasopoulos

**Affiliations:** 1 Medical School, University of Oxford, Oxford OX3 9BL, UK; 2 Department of Rheumatology, Oxford University Hospitals NHS Foundation Trust, Oxford OX3 9DU, UK; 3 Nuffield Department of Orthopaedics Rheumatology and Musculoskeletal Sciences, University of Oxford, Oxford OX3 7LD, UK; 4 Department of Cardiac Surgery, Oxford University Hospitals NHS Foundation Trust, Oxford OX3 9DU, UK; 5 Department of Radiology, Oxford University Hospitals NHS Foundation Trust, Oxford OX3 9DU, UK; 6 Department of Vascular Surgery, Oxford University Hospitals NHS Foundation Trust, Oxford OX3 9DU, UK

**Keywords:** cardiac surgery, aorta, aortitis, aortic aneurysm, aortic dissection, vasculitis, re-operations

## Abstract

Aortitis, defined as inflammation of the aorta, can lead to aneurysms and dissections. Intra-operative sampling is essential for diagnosis, with many cases presenting asymptomatically as clinically isolated aortitis. Previous studies investigating aortitis in major aortic surgery have been limited by low intra-operative sampling. We performed an 11-year, retrospective, cross-sectional study to investigate the true prevalence of aortitis in thoracic aortic aneurysms and dissections by analysing all major aortic operations performed in a single centre. We collected medical histories, histological reports, post-operative outcomes and follow-up data; 537 patients met the inclusion criteria, representing an 88% histological sampling rate. The prevalence of aortitis was 10.6% (*n* = 57), of which 75% were clinically isolated. The re-operation rate in aortitis was twice that of non-aortitis patients (17.5% vs. 9.4%, *p* = 0.054). Multivariate logistic regression identified increased age, female sex, current smoking, and other inflammatory diseases as significantly associated with aortitis, with a bicuspid aortic valve associated with a significantly decreased likelihood of aortitis. The true prevalence of aortitis is likely higher than reported in previous studies, with our study showing twice the prevalence found in previous studies with lower sampling rates. Due to the increased re-intervention in aortitis, specialist multi-disciplinary follow-up and aortitis centres should be formed.

## 1. Introduction

Aortitis, which is defined as inflammation of the aorta [1,2,3], can be classified aetiologically as infectious and non-infectious. Non-infectious aortitis can be further subclassified as inflammatory or clinically isolated aortitis (CIA) [1,2,3]. Infectious aortitis is a rare form of aortitis caused by a range of bacterial organisms, including Salmonella species and Staphylococcal species [1]. Inflammatory aortitis is commonly caused by large vessel vasculitides such as Giant Cell Arteritis (GCA) and Takayasu’s arteritis (TA) and is also associated with several other inflammatory conditions such as Rheumatoid Arthritis, Cogan’s syndrome and Behçet’s disease [1]. CIA is defined as aortitis in the absence of systemic vasculitis or inflammatory conditions [2,3,4]. CIA is technically a subtype of inflammatory aortitis but is often grouped separately due to the lack of clarity about pathophysiology and outcomes and typically does not have too many symptoms. CIA is reported to represent the largest group of post-surgically diagnosed aortitis and among all aortitis in cardiac aortic surgery for aortic aneurysms and type A aortic dissections, with a reported prevalence between 60 and 90% of all aortitis [5,6,7,8].

**Figure 1:**
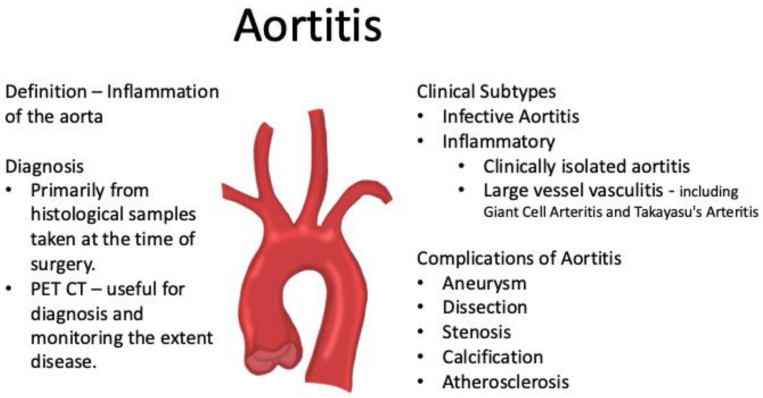
Characteristic summary of aortitis.

The clinical presentation of aortitis is highly variable, making diagnosis challenging [1,9]. Patients classically present with high inflammatory markers along with non-specific symptoms such as fatigue, feeling generally ill, weight loss or polymyalgic symptoms with proximal stiffness around the shoulder and pelvic girdle [3]. Aortitis is therefore clinically diagnosed following a thorough medical history, blood tests and positron emission tomography-computed tomography (PET-CT). Aortitis can lead to aortic wall thickening, loss of vascular elasticity, stenosis and occlusion. It can also present initially as aortic aneurysms and/or dissections, including type A aortic dissections (TAAD), which are managed surgically [6,9,10,11]. Aortic aneurysms and dissections can be diagnosed through a variety of imaging techniques, but principal initial investigations are with echocardiography and computed tomography (CT) scans [12]. In patients undergoing cardiac surgery for aneurysms and dissections of the aorta, diagnosis of aortitis can be made by histological sampling of samples resected intra-operatively. Diagnosis remains important even after surgical repair as it can increase the risk of post-operative complications and the development of new aneurysms, which necessitate further operations [11,13]. Prompt referral to specialist rheumatology for commencement of anti-inflammatory therapies is essential with the aim of preventing future vascular complications. In patients who have been diagnosed with aortitis, aneurysms and dissections tend to occur more frequently, suggesting that appropriate treatment with disease-modifying drugs might be able to reduce the risk of aneurysm development and hence the need for major aortic surgery [9] (Figure 1).

Whilst the prevalence and impact of inflammatory aortitis on aneurysm formation are well documented [6,13,14], the prevalence and clinical significance of CIA in cardiac surgery are unclear [3,11,15]. Many studies in cardiac surgery have reported a prevalence of all-type aortitis between 3 and 6% [5,6,7,8,11] and the prevalence of CIA at 4–5% [5,6,7,8,10,11]. However, many of these studies fail to accurately report sampling methodologies or are based on pathology records, creating selection bias [6,7,8,11]. Our literature review has revealed only two studies which clearly reported the number of pathology reports based on aortic sampling at the time of surgery (with a sampling rate of 50% [5] and 97% [10]). It is therefore likely that the prevalence of aortitis in cardiac surgery is higher than reported, which means the true prevalence of CIA is essentially unknown.

We aimed to investigate the true prevalence of aortitis in patients undergoing surgery for aortic aneurysms and TAAD, identify any potential risk factors for aortitis and the impact on long-term outcomes.

## 2. Materials and Methods

### 2.1. Ethical Statement

This study was carried out in accordance with the Declaration of Helsinki. All patients included in this study provided written informed consent to the use of their data in clinical audits and research. Human ethics was not required. This study was approved by Oxford University Hospitals NHS Foundation Trust (Audit Number: 8036).

### 2.2. Study Design

This is a retrospective cohort study of prospectively collected data. We reviewed all major aortic operations performed at Oxford University Hospitals NHS Foundation Trust, United Kingdom, over an 11-year period (1/1/2012 to 31/12/2022). Major aortic operations were defined as all operations on TAAD and surgical repairs for aneurysms of the root, ascending and/or arch of the aorta performed by cardiac surgeons. Thoracoabdominal aneurysms were not included as these operations are not performed at our centre. The inclusion criteria for this study were all major aortic operations with histological sampling of the aorta performed at our centre. The primary aim was to investigate the prevalence of aortitis in patients undergoing major aortic surgery performed by cardiac surgeons. The secondary aims were to identify any potential risk factors for aortitis and investigate the impact of aortitis on long-term outcomes.

### 2.3. Data Collection and Sorting

All cardiothoracic operations at the trust are stored in a database, and an extract was created for all patients who had consented to the use of their information for audit and research. The database contained their age at surgery, date of operation and cardiovascular risk factors. Medical notes were reviewed using the local electronic patient record system. This collected operation notes, the major indication for surgery, past medical history, presence of infective endocarditis, aortic valve morphology, histological report of the aorta, post-operative outcomes and requirement for further surgery. Re-operations were defined as all patients who had a re-operation, patients who were listed for a re-operation but at the point of data collection had not yet received it, and patients who required it but were medically unfit. Re-operations excluded operations in the immediate post-operative period due to bleeding and surgical site infections.

Initial sorting was performed to ensure the classifications of risk factors were correct. The pre-operative characteristics in Table 1 are defined as the following: Arteritis is a previous rheumatological diagnosis of an arteritis in a vascular bed other than the aorta [3]. Our cohort contained GCA, TA and ANCA-associated vasculitis. Other inflammatory conditions included inflammatory and autoimmune diseases, which are associated with an increased risk of cardiovascular events such as Crohn’s disease, polymyalgia rheumatica, systemic lupus erythematous and Rheumatoid Arthritis [16]. Clinical diagnoses were collected, and ACR/EULAR 2022 classification criteria for GCA and TA were retrospectively applied to reclassify these patients as GCA and TA [17,18]. Connective tissue diseases included Marfan’s Syndrome, Loez-Dietz Syndrome, and other confirmed hereditary thoracic aortic diseases.

Aortitis was defined as when samples of the aorta resected at the time of surgery were sent for histological analysis and demonstrated aortitis [2]. This is referred to as histological sampling or biopsies within the manuscript. Aortitis was subclassified as inflammatory, infectious or CIA. Inflammatory aortitis is aortitis in the presence of a vasculitis in another vascular bed or rheumatological inflammatory conditions such as Rheumatoid Arthritis. CIA is aortitis in the absence of inflammatory or infective aortitis.

Nine patients underwent major aortic surgery prior to 2012 and received further major aortic surgery within the time frame we investigated. Two of these patients were diagnosed with clinically isolated aortitis at the operation within the time frame investigated. None of these patients had a previous diagnosis of vasculitis, aortitis or other rheumatological condition. This cohort was included as they provide valuable information on long-term outcomes in major aortic surgery. Their age and date of operation were corrected for their first major aortic operation. If only the year of the previous surgery was available, then it was recorded as the first of June of the given year.

### 2.4. Statistical Analysis

Statistical analysis was performed on Microsoft Excel and R using RStudio (Version 2022.07.2).

Variables were checked for normal distributions. Descriptive statistics were employed to summarise patient demographics and indications for re-operations. Fisher’s exact test was used to compare the subgroups of re-operations between those with and without aortitis. Fisher’s exact test was also used to compare the re-operations and death between CIA and inflammatory aortitis against no aortitis.

Kaplan–Meir curves were generated to investigate the timing of re-operation due to aortic aneurysms between patients with and without aortitis. Log-rank testing was used to investigate statistical significance.

Logistic regression investigated risk factors for histologically proven aortitis. Due to incomplete reporting of cardiovascular risk factors, 123 patients were excluded from the regression analysis. Prior to running logistic regression, predictors were included if they were present in more than 2.5% of the patients to ensure the features truly explain the dataset. Variables were grouped as necessary. Residual diagnostic regression testing was conducted for the primary outcomes. The linearity of continuous variables with the outcomes was assessed using fractional polynomials, and collinearity between variables was assessed using the variance inflation factor (VIF).

## 3. Results

**Figure 2:**
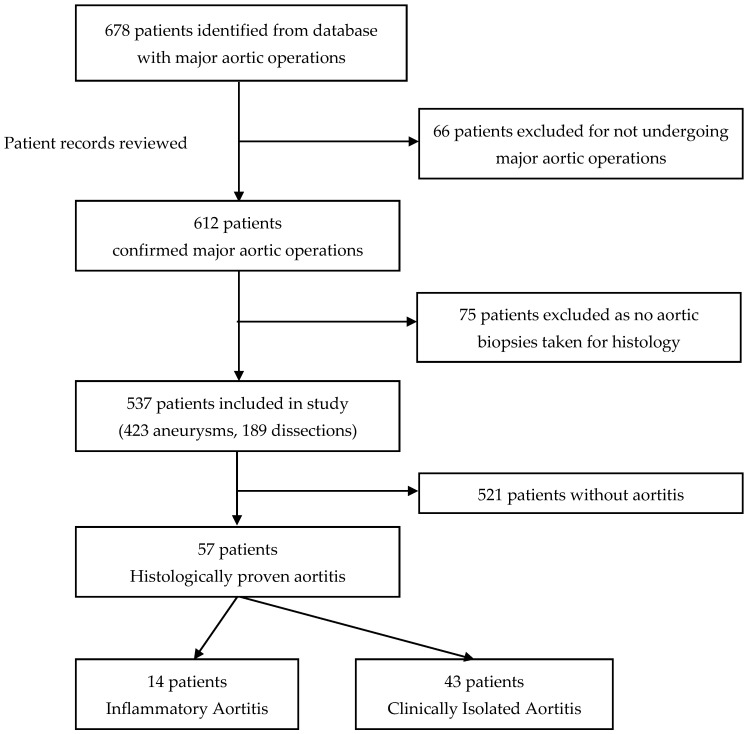
Consort diagram of patient selection.

A cohort of 690 procedures logged as major aortic operations performed on 678 patients were identified from the database. Of those, 612 patients were confirmed to have received a major aortic operation (423 aneurysms and 189 dissections). Seventy-five patients were excluded as they were missing histological reports (Figure 2).

A total of 537 patients met the inclusion criteria, with a median follow-up time of 4.08 years (inter-quartile range 1.69–7.19), with 32.2% having undergone surgery for TAAD. Patient demographics included in the study are summarised in Table 1. The median age was 63 years old, and most patients were male (68%).

**Table 1:** Patient demographics.

Patient Demographics	Clinically Isolated Aortitis (*n* = 43)	Inflammatory Aortitis (*n* = 14)	No Aortitis (*n* = 480)
Age	71 (62, 76) ^1^	73 (68, 77) ^1^	62 (51, 71) ^1^
Male Sex	15 (35%)	2 (14%)	347 (72%)
BMI	24.3 (22.8–25.7) ^1^ *n* = 38	25.8 (24.2–30.1) ^1^	27.0 (23.9-30.9) ^1^ *n* = 423
Hypercholesterolemia	8/40 (20%)	4 (29%)	141/424 (33%)
Diabetes	0/42 (0%)	1 (7.1%)	19/473 (4.0%)
Hypertension	32/39 (82%)	8/13 (62%)	303/464 (65%)
Smoking Status			
Current smoker	7/40 (18%)	3 (21%)	31/429 (7.2%)
Ex-smoker	14/40 (35%)	4 (29%)	145/429 (34%)
Never smoked	19/40 (48%)	7 (50%)	253/429 (59%)
Previous Cardiac Surgery	1 (2.3%)	0 (0%)	32 (6.7%)
Bicuspid Aortic Valve	4 (9.3%)	2 (14%)	175 (36%)
Previous Type B Dissection	1 (2.3%)	0 (0%)	8 (1.7%)
Connective Tissue Disease	2 (4.7%)	0 (0%)	31 (6.5%)
Arteritis	0 (0%)	7 (50%)	4 (0.8%)
Other Inflammatory Condition	0 (0%)	7 (50%)	9 (1.9%)
Previous Chemotherapy or Radiotherapy	0 (0%)	0 (0%)	4 (0.8%)

^1^ Median (IQR); *n* (%). BMI, hypercholesterolaemia, diabetes and smoking status were not reported in some patients, these patients were removed for those columns, and the sample size included is reported in each column.

Eighty-eight per cent of all major aortic operations had histological sampling performed (*n* = 537), of which 10.6% of patients (*n* = 57) had biopsy-proven aortitis; 8.0% (*n* = 43) had CIA; 2.6% (*n* = 14) had inflammatory aortitis; and no patients had infectious aortitis. Of the patients with inflammatory aortitis, eight patients were classified as GCA, one as TA, two had historic diagnoses of polymyalgia rheumatica, and three had Rheumatoid Arthritis (two patients had seronegative Rheumatoid Arthritis, and one patient was not specified).

**Figure 3:**
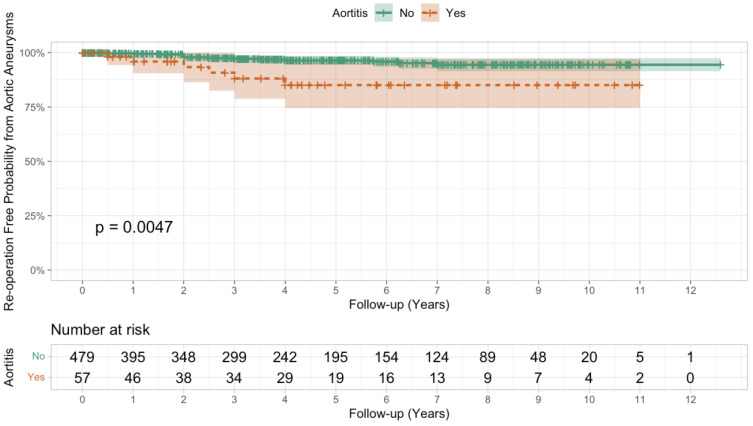
Kaplan–Meir analysis of re-operations due to aortic aneurysms for patients with and without aortitis. Log-rank testing was performed.

The re-operation rate for the non-aortitis cohort was 9.4% (*n* = 45/480), but the re-operation rate in the all-type aortitis group was double, 18% (*n* = 10/57), nearing statistical significance (Pearson’s chi-squared test, *p* = 0.054). When subtype analysis was performed, patients with inflammatory aortitis were at particularly increased risk of re-operation with a 29% re-operation rate (Fisher’s exact test, P = 0.041 in comparison to no aortitis), vs. a 14% re-operation rate for CIA (not statistically significant) (Table 2). The indications for re-operation were reviewed, and whilst there was a wide range of indications for re-operation in the no aortitis group, re-operations due to aneurysms of any segment of the aorta are over-represented in the aortitis group (Table 3). Odds ratio analysis for the indication for re-operation was limited due to the small size, but aortitis led to a statistically significant increase in the risk of re-operation due to new aneurysm formation (Table 3). The risk of new aneurysm formation in aortic dissections was higher (3/15 with aortitis vs. 10/158 without) in comparison to aortic aneurysm as the initial pathology (5/42 with aortitis vs. 8/322 without). Kaplan–Meir analysis of re-operations due to aortic aneurysms demonstrated that patients with aortitis are at increased risk of re-operation in comparison to patients without aortitis (*p* = 0.0047, log-rank test) (Figure 3).

### Risk Factors for Aortitis

Logistic regression was performed to investigate risk factors for aortitis in major aortic operations (Table 4). Four hundred fourteen patients were included in the regression analysis as they had complete cardiovascular risk factor collection. Multivariate logistic regression identified increased age, female sex, current smokers and inflammatory diseases as associated with increased risk of aortitis, whilst hypercholesterolaemia and a bicuspid aortic valve are associated with decreased risk of aortitis.

## 4. Discussion

### 4.1. Prevalence of Aortitis

In our study, the prevalence of all group aortitis was 10.6%, with an 8.0% prevalence of CIA. This is higher than reported in the literature [5,6,10,11,14]. Whilst the prevalence of inflammatory aortitis was similar to other studies, the prevalence of CIA was almost double what has previously been reported [5,6,10,11,14]. While one study reported a sampling rate of 97% and a prevalence of CIA of 3.8%, it only had 206 patients [10]. This disparity may be due to our relatively high histological sampling rate. Previous reports had differing methodologies where patients were identified through analysed histological samples and did not report how many patients did not have sampling performed [6,11,14].

### 4.2. Risk Factors for Aortitis

Our study identified previous inflammatory diseases (OR 9.01, *p* < 0.001), female sex (OR 4.10, *p* < 0.001) and current smokers (OR 3.43, *p* = 0.036) as associated with increased risk of developing aortitis, whilst increased age had a small effect (OR1.03, *p* = 0.040) (Table 4). Bicuspid aortic valve (OR 0.34, *p* = 0.036) and hypercholesterolaemia (OR 0.39, *p* = 0.028) were associated with a reduced risk of aortitis (Table 4). The association of large vessel vasculitis with female sex, which was reported in our study, has been well documented in other studies [19]. Other studies have identified diabetes and connective tissue disease as associated with aortitis [5]; however, this was not found in our study. Diabetes and connective tissue disease both had a low prevalence in our study, which may account for this. Some other studies have also reported a negative association of diabetes with GCA [20]. The effect of a bicuspid aortic valve on aortitis has not been reported before, and the reasons for the decreased risk of aortitis in a bicuspid aortic valve are unclear.

### 4.3. Long-Term Outcomes of Aortitis

Aortitis can lead to several vascular complications, including vascular stenosis, increased risk of stroke and increased risk of aneurysm formation [13]. In our study, patients with aortitis are at a much-increased risk of re-operation, specifically due to new aneurysm formation (Table 3, Figure 3). This is important as patients with aortitis will require additional close monitoring with cross-sectional imaging to identify new aneurysm formation. Inflammatory aortitis presents a clear increased risk which needs to be monitored (Table 2). The impact of CIA aortitis remains unclear, with a small, non-statistically significant increase in the risk of re-operation (Table 2). We did not assess radiological data, but another study reported that 17% of CIA developed new aneurysms [11], which is in keeping with the 12% re-operation rate in our study (Table 2). In contrast, other studies have reported a high complication rate in CIA; the French aortitis group reported a 58% 5-year vascular complication rate [21,22]. It therefore remains essential to biopsy every patient undergoing surgery for aneurysms and dissections of the aorta to allow the identification of aortitis and prevent any sampling bias in the histological samples that are being performed.

### 4.4. Management of Aortitis

Historically, patients who underwent cardiac surgery for aortic aneurysms and dissections and were found to have aortitis were followed up in the standard cardiac surgery clinic and referred to their local rheumatology unit. In reflection of this rare and highly complicated cohort, this is unlikely to be in the patient’s best interest. We recommend that aortic centres develop dedicated centralised services to follow-up these patients in a multi-disciplinary environment where rheumatology, cardiac surgery, interventional radiology and vascular surgery can collaborate and manage the multifaceted aspects of these conditions effectively. In our centre, aortitis patients now receive enhanced surgical follow-up in a dedicated complex aortic clinic with subspecialist input from aortitis specialist rheumatologists. Upon diagnosis of aortitis, all patients receive a rheumatological review in their rapid assessment vasculitis clinic with blood tests, including a full screen of inflammatory and infective markers (ESR, CRP and syphilis serology), PET-CT to assess the extent of inflammation and annual CT aortogram for at least 5 years.

The medical management of patients with aortitis within our unit has been described previously [9]. First-line management of aortitis was with corticosteroids such as Prednisolone (0.75–1 mg/kg) alongside conventional synthetic disease-modifying agents such as Methotrexate (20–25 mg/week) [23]. Methotrexate was usually started together with corticosteroids to mitigate the long-term side effects of corticosteroids and the risk of iatrogenic Addison’s disease. In refractory cases, more potent treatments such as Cyclophosphamide are used (15 mg/kg intravenous in pulsed regimes). Tocilizumab (162 mg subcutaneous weekly), an interleukin-6 inhibitor, was successfully proven to be effective in GCA [24] and is available in the UK for use in aortitis in ‘relapsing and refractory cases’, following the failure of two conventional treatment strategies [25]. Most of the therapies used in large vessel vasculitis are T-cell-based due to the immunological phenotype. The Oxford aortitis study (which included all types of aortitis) has shown that these patients need therapy for several years, with a median duration of nearly 72 months; nonetheless, some patients had come off therapy successfully without flares of aortitis [9]. In addition, secondary prevention therapies, including statins, beta-blockers, ACE inhibitors and anticoagulants/antiplatelets are used to cater to the individual needs of each patient to optimise their cardiovascular risk factors. It is therefore important that therapeutic management of patients with aortitis incorporates both monitoring for cardiovascular complications as well as the use of anti-inflammatory treatments where appropriate.

### 4.5. Future Challenges in the Management of Aortitis

Aortitis patients remain at high risk of complications (Table 2), requiring medical and surgical follow-up. We recommend the creation of centralised aortitis centres that can provide subspecialty therapies, enhanced surveillance for complications, and allow further research into the field of aortitis. At present, there are no clinical trials that specifically investigate the management of aortitis, and the optimum treatment remains a challenging aspect, even for established vasculitis specialists. Although biologics are now available, significant restrictions remain on their use (particularly in the United Kingdom), and the results from conventional disease-modifying drugs in reducing complications do not appear to be very encouraging [9]. CIA represents a particular challenge as to whether they should be managed as a distinct group of aortitis or not [15,26].

### 4.6. Limitations

This was a retrospective cohort study. Some data were incomplete, such as histological sampling of the aorta and incomplete collecting of cardiovascular risk factors, which resulted in patients being excluded from the study and regression analysis, respectively. Due to the relatively small size of the aortitis cohort, it was not possible to investigate the effect of medical treatment of aortitis on surgical outcomes. A review of the histological subtype was not possible as microscope slides are stored off-site from the hospital and are not available in a digital format for review.

## 5. Conclusions

**Figure 4:**
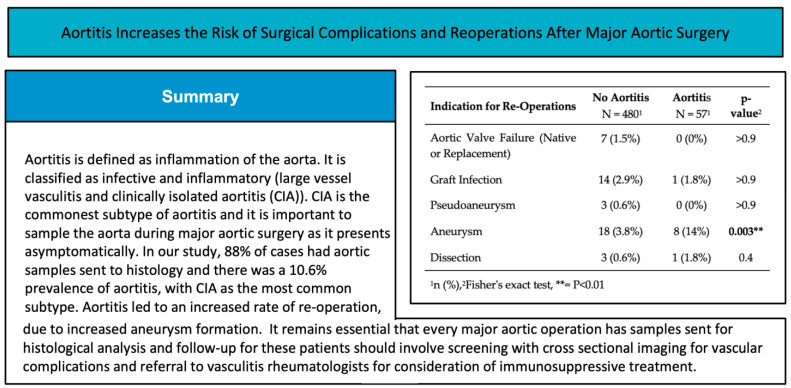
Central figure.

Aortitis is a rare but important cause of aortic aneurysms and dissections. Intra-operative sampling is essential to identify aortitis. Aortitis leads to an increased risk of further aortic aneurysms, which necessitates enhanced and prolonged follow-up for all diagnosed patients who have undergone major aortic surgery. Due to the rarity of the condition, specialist aortitis centres should be developed to allow a multi-disciplinary approach and aid further research into the field of aortitis (Figure 4).

## Figures and Tables

**Table 2:** Re-operations in major aortic surgery.

Type of Aortitis	Re-Operation Rate
CIA	6 (14%, *n* = 43)
Inflammatory Aortitis	4 (29%, *n* = 14) *
No Aortitis	45 (9.4% *n* = 480)

*n* (%), * *p* < 0.05 Fisher’s Exact Test vs. No Aortitis.

**Table 3:** Indications for re-operations.

Indication for Re-Operations	No Aortitis *n* = 480	Aortitis *n* = 57	*p*-Value ^1^
Aortic Valve Failure (Native or Replacement)	7 (1.5%)	0 (0%)	>0.9
Graft Infection	14 (2.9%)	1 (1.8%)	>0.9
Pseudoaneurysm	3 (0.6%)	0 (0%)	>0.9
Aneurysm	18 (3.8%)	8 (14%)	0.003 **
Dissection	3 (0.6%)	1 (1.8%)	0.4

*n* (%), ^1^ Fisher’s exact test, ** = *p* < 0.01.

**Table 4:** Logistic regression for predictors of aortitis.

		Univariate		Multivariate		
Predictor	OR ^1^	95% CI ^1^	*p*-Value	OR ^1^	95% CI ^1^	*p*-Value
Age	1.05	1.02, 1.08	<0.001 ***	1.03	1.00, 1.07	0.040 *
Female Sex	6.13	3.20, 12.30	<0.001 ***	4.10	1.96, 8.89	<0.001 ***
BMI	0.95	0.89, 1.02	0.170	1.01	0.93, 1.09	0.9
Hypercholesterolaemia	0.69	0.33, 1.35	0.302	0.39	0.16, 0.87	0.028 *
Diabetes	0.49	0.03, 2.48	0.492	0.74	0.04, 4.79	0.8
Hypertension	1.85	0.92, 4.05	0.099	1.33	0.53, 3.57	0.6
Previous Smoker	1.15	0.59, 2.18	0.065	1.70	0.77, 3.75	0.2
Current Smoker	3.13	1.36, 6.75	0.004 **	3.43	1.18, 9.67	0.020 *
Bicuspid Aortic Valve	0.21	0.08, 0.48	<0.001 ***	0.34	0.12, 0.88	0.036 *
Connective Tissue Disease	0.62	0.10, 2.19	0.530	1.26	0.17, 6.27	0.8
Inflammatory Diseases	9.64	4.04, 22.93	<0.001 ***	9.01	3.16, 26.3	<0.001 ***

^1^ OR = Odds Ratio, CI = Confidence Interval, * = *p* < 0.05, ** = *p* < 0.01, *** = *p* < 0.001.

## Data Availability

Data are available upon request.

## References

[1] Gornik H.L., Creager M.A. (2008). Aortitis. Circulation.

[2] Stone J.R., Bruneval P., Angelini A., Bartoloni G., Basso C., Batoroeva L., Buja L.M., Butany J., d’Amati G., Fallon J.T. (2015). Consensus statement on surgical pathology of the aorta from the Society for Cardiovascular Pathology and the Association for European Cardiovascular Pathology: I. Inflammatory diseases. Cardiovasc. Pathol..

[3] Pugh D., Grayson P., Basu N., Dhaun N. (2021). Aortitis: Recent advances, current concepts and future possibilities. Heart.

[4] Cinar I., Wang H., Stone J.R. (2017). Clinically isolated aortitis: Pitfalls, progress, and possibilities. Cardiovasc. Pathol..

[5] Schmidt J., Sunesen K., Kornum J.B., Duhaut P., Thomsen R.W. (2011). Predictors for pathologically confirmed aortitis after resection of the ascending aorta: A 12-year Danish nationwide population-based cross-sectional study. Arthritis Res. Ther..

[6] Pacini D., Leone O., Turci S., Camurri N., Giunchi F., Martinelli G.N., Di Bartolomeo R. (2008). Incidence, etiology, histologic findings, and course of thoracic inflammatory aortopathies. Ann. Thorac. Surg..

[7] Espitia O., Bruneval P., Assaraf M., Pouchot J., Liozon E., de Boysson H., Gaudric J., Chiche L., Achouh P., Roussel J.-C. (2023). Long-Term Outcome and Prognosis of Noninfectious Thoracic Aortitis. J. Am. Coll. Cardiol..

[8] Clifford A.H., Arafat A., Idrees J.J., Roselli E.E., Tan C.D., Rodriguez E.R., Svensson L.G., Blackstone E., Johnston D., Pettersson G. (2019). Outcomes Among 196 Patients with Noninfectious Proximal Aortitis. Arthritis Rheumatol..

[9] Ahmad N., Andev R., Verdiyeva A., Dubey S. (2023). Single centre experience of 120 patients with non-infectious aortitis: Clinical features, treatment and complications. Autoimmun. Rev..

[10] Kaymakci M., Elfishawi M., Langenfeld H.E., Crowson C.S., Weyand C.M., Koster M.J., Warrington K.J. (2023). The epidemiology of pathologically confirmed clinically isolated aortitis: A North American population-based study. Clin. Exp. Rheumatol..

[11] Rojo-Leyva F., Ratliff N.B., Cosgrove D.M., Hoffman G.S. (2000). Study of 52 patients with idiopathic aortitis from a cohort of 1204 surgical cases. Arthritis. Rheum..

[12] Perone F., Guglielmo M., Coceani M., La Mura L., Dentamaro I., Sabatino J., Gimelli A. (2023). The Role of Multimodality Imaging Approach in Acute Aortic Syndromes: Diagnosis, Complications, and Clinical Management. Diagnostics.

[13] Gaudric J., Dennery M., Jouhannet C., Kagan N., Saadoun D., Chiche L., Koskas F. (2016). Aortitis and surgery. Rev. Med. Interne.

[14] Miller D.V., Isotalo P.A., Weyand C.M., Edwards W.D., Aubry M., Tazelaar H.D. (2006). Surgical pathology of noninfectious ascending aortitis: A study of 45 cases with emphasis on an isolated variant. Am. J. Surg. Pathol..

[15] Stone J.R. (2023). The Winding Path Toward Understanding Clinically Isolated Aortitis. J. Am. Coll. Cardiol..

[16] Sorriento D., Iaccarino G. (2019). Inflammation and Cardiovascular Diseases: The Most Recent Findings. Int. J. Mol. Sci..

[17] Grayson P.C., Ponte C., Suppiah R., Robson J.C., Gribbons K.B., Judge A., Craven A., Khalid S., Hutchings A., Danda D. (2022). 2022 American College of Rheumatology/EULAR classification criteria for Takayasu arteritis. Ann. Rheum. Dis..

[18] Ponte C., Grayson P.C., Robson J.C., Suppiah R., Gribbons K.B., Judge A., Craven A., Khalid S., Hutchings A., Watts R.A. (2022). 2022 American College of Rheumatology/EULAR Classification Criteria for Giant Cell Arteritis. Arthritis Rheumatol..

[19] Saadoun D., Vautier M., Cacoub P. (2021). Medium- and Large-Vessel Vasculitis. Circulation.

[20] Mukhtyar C., Myers H., Jones C., Dhatariya K. (2020). The relationship between glycated haemoglobin levels and the risk of giant cell arteritis—A case–control study. Rheumatol. Adv. Pract..

[21] Ferfar Y., Morinet S., Espitia O., Agard C., Vautier M., Comarmond C., Desbois A.C., Domont F., Resche-Rigon M., Cacoub P. (2021). Spectrum and Outcome of Noninfectious Aortitis. J. Rheumatol..

[22] Wang H., Smith R.N., Spooner A.E., Isselbacher E.M., Cambria R.P., MacGillivray T.E., Stone J.H., Stone J.R. (2012). Giant cell aortitis of the ascending aorta without signs or symptoms of systemic vasculitis is associated with elevated risk of distal aortic events. Arthritis Rheum..

[23] Hellmich B., Agueda A., Monti S., Buttgereit F., de Boysson H., Brouwer E., Cassie R., Cid M.C., Dasgupta B., Dejaco C. (2020). 2018 Update of the EULAR recommendations for the management of large vessel vasculitis. Ann. Rheum. Dis..

[24] Stone J.H., Tuckwell K., Dimonaco S., Klearman M., Aringer M., Blockmans D., Brouwer E., Cid M.C., Dasgupta B., Rech J. (2017). Trial of Tocilizumab in Giant-Cell Arteritis. N. Engl. J. Med..

[25] NHS England Clinical Commissioning Policy: Tocilizumab for Takayasu Arteritis (Adults) 2016. https://www.england.nhs.uk/wp-content/uploads/2018/07/Tocilizumab-for-Takayasu-arteritis.pdf.

[26] Mayer A., Sperry A., Quimson L., Rhee R.L. (2022). Long-Term Clinical and Radiographic Outcomes in Patients with Clinically Isolated Aortitis. ACR Open Rheumatol..

